# Chatbot-Delivered Cognitive Behavioral Therapy in Adolescents With Depression and Anxiety During the COVID-19 Pandemic: Feasibility and Acceptability Study

**DOI:** 10.2196/40242

**Published:** 2022-11-22

**Authors:** Ginger Nicol, Ruoyun Wang, Sharon Graham, Sherry Dodd, Jane Garbutt

**Affiliations:** 1 Division of Child and Adolescent Psychiatry Department of Psychiatry Washington University School of Medicine St Louis, MO United States; 2 Division of Allergy, Immunology & Pulmonology Department of Pediatrics Washington University School of Medicine St Louis, MO United States

**Keywords:** COVID-19, adolescent depression, mobile health, cognitive behavioral therapy, chatbot, relational conversational agent, depression, anxiety, suicide, self-harm, pandemic, pediatric, youth, adolescent, adolescence, psychiatry, conversational agent, CBT, clinic, data, acceptability, feasibility, usability, primary care, intervention, mental health, digital health, technology mediated, computer mediated

## Abstract

**Background:**

Symptoms of depression and anxiety, suicidal ideation, and self-harm have escalated among adolescents to crisis levels during the COVID-19 pandemic. As a result, primary care providers (PCPs) are often called on to provide first-line care for these youth. Digital health interventions can extend mental health specialty care, but few are evidence based. We evaluated the feasibility of delivering an evidence-based mobile health (mHealth) app with an embedded conversational agent to deliver cognitive behavioral therapy (CBT) to symptomatic adolescents presenting in primary care settings during the pandemic.

**Objective:**

In this 12-week pilot study, we evaluated the feasibility of delivering the app-based intervention to adolescents aged 13 to 17 years with moderate depressive symptoms who were treated in a practice-based research network (PBRN) of academically affiliated primary care clinics. We also obtained preliminary estimates of app acceptability, effectiveness, and usability.

**Methods:**

This small, pilot randomized controlled trial (RCT) evaluated depressive symptom severity in adolescents randomized to the app or to a wait list control condition. The primary end point was depression severity at 4-weeks, measured by the 9-item Patient Health Questionnaire (PHQ-9). Data on acceptability, feasibility, and usability were collected from adolescents and their parent or legal guardian. Qualitative interviews were conducted with 13 PCPs from 11 PBRN clinics to identify facilitators and barriers to incorporating mental health apps in treatment planning for adolescents with depression and anxiety.

**Results:**

The pilot randomized 18 participants to the app (n=10, 56%) or to a wait list control condition (n=8, 44%); 17 participants were included in the analysis, and 1 became ineligible upon chart review due to lack of eligibility based on documented diagnosis. The overall sample was predominantly female (15/17, 88%), White (15/17, 88%), and privately insured (15/17, 88%). Mean PHQ-9 scores at 4 weeks decreased by 3.3 points in the active treatment group (representing a shift in mean depression score from moderate to mild symptom severity categories) and 2 points in the wait list control group (no shift in symptom severity category). Teen- and parent-reported usability, feasibility, and acceptability of the app was high. PCPs reported preference for introducing mHealth interventions like the one in this study early in the course of care for individuals presenting with mild or moderate symptoms.

**Conclusions:**

In this small study, we demonstrated the feasibility, acceptability, usability, and safety of using a CBT-based chatbot for adolescents presenting with moderate depressive symptoms in a network of PBRN-based primary care clinics. This pilot study could not establish effectiveness, but our results suggest that further study in a larger pediatric population is warranted. Future study inclusive of rural, socioeconomically disadvantaged, and underrepresented communities is needed to establish generalizability of effectiveness and identify implementation-related adaptations needed to promote broader uptake in pediatric primary care.

**Trial Registration:**

ClinicalTrials.gov NCT04603053; https://clinicaltrials.gov/ct2/show/NCT04603053

## Introduction

Depression and anxiety are common in adolescents. [1,2]. If left untreated, these common illnesses can result in significant sequelae including school dropout, substance abuse, and suicide [3]. At any one time, 18% of adolescents report depressive symptoms [1,2], and about 3% are diagnosed with a depressive disorder [2]. Depression and anxiety often co-occur; 1 in 3 teens will experience clinically significant anxiety symptoms [4]. These numbers have risen in recent years due to the isolating effects of quarantine and the stress of virtual learning during the COVID-19 pandemic [5,6]. Even before the pandemic, suicide was the second highest cause of death in this age group [7-9], but in 2020, emergency department (ED) visits for suspected suicide attempts increased by 30% compared to 2019 [10]. The effects of the pandemic on mental health in general has been unprecedented; prepandemic mental health workforce shortages have become more dire. Thus, there is an urgent need to provide prompt, effective treatments that can be readily integrated into the day-to-day lives of adolescents and disseminated remotely during periods of quarantine and ongoing workforce issues.

Evidence-based treatments for pediatric depression and anxiety include selective serotonin and serotonin-norepinephrine reuptake inhibitor (SSRI, SNRI) medications and cognitive behavioral therapy (CBT), used separately or in combination [11-13]. Yet, few teens receive treatment with these therapies [13]. CBT is typically provided through a series of face-to-face interactions with a trained therapist over several months and is often preferred by families over medication [3,14]. However, CBT is seldom used due to pervasive and persistent problems with access, cost, and stigma [15-18]. Internet and mobile health (mHealth) interventions reduce these barriers to utilization and are acceptable and effective alternatives to live CBT for the treatment of depression and anxiety in adults [19]. Conversational agents or “chatbots” that deliver CBT via a text-based, semiautomated algorithm are known to reduce mild-to-moderate depressive symptoms in nonclinical populations [17,20-23]. However, few have been evaluated in clinical populations, with scarce rigorous study in youth [21,24,25].

About 95% of teens in the United States use smartphones [26] or other web-enabled mobile devices, and nearly half report being online almost constantly. Over half of US teens use their devices for accessing social media platforms, and this number may be higher in adolescents with known mental health diagnoses [27]. Although internet use—social media use in particular—has known serious negative effects on adolescent mental health [28], mobile technology can also promote social support and even assistance to young people dealing with emotional challenges [29]. Adolescents are increasingly using their devices to search the internet for information about mental health [18]. Many teens want to be self-reliant when coping with emotional distress and prefer to manage difficult situations virtually rather than through in-person interactions [18]. However, few digital therapeutic interventions have been specifically developed for children and adolescents [26]. Using mHealth to deliver CBT to teens merits further evaluation, as it is inexpensive, easy to access and administer, and scalable, which are important attributes for widespread dissemination, adoption, and public health impact [30].

Because the management of adolescents with depression and anxiety is often done by primary care providers (PCPs), the goal of this study was to investigate the feasibility of implementing an existing mHealth intervention [20,31] as part of the treatment plan for adolescents diagnosed with depression and anxiety by their PCP. The intervention, Woebot for Adolesent Depression, or W-GenZ, is an mHealth application that uses a relational conversational agent known as Woebot to deliver CBT to adolescents presenting with mood and anxiety concerns. W-GenZ is one among a suite of emotional support Woebot-based applications that have been previously studied. Previous research demonstrated Woebot’s feasiblity and acceptability as well as efficacy to reduce (1) depression and anxiety among young adults [20], (2) depression among postpartum women [32,33], and (3) substance abuse among adults [34,35]. In addition, data indicate that Woebot establishes a therapeutic working alliance with users [33]. The W-GenZ app was developed specifically for use in adolescents, and while preliminary effectiveness data among this population are promising [36], it has not yet been rigorously tested for effectiveness. Moreover, the application has not yet been implemented in real-world clinical settings, where such interventions would ideally track and report progress to PCPs and parents, which is critical for maintaining safety and treatment adherence.

In this open-label, randomized pilot study, our objectives were 2-fold: (1) to establish that the app is usable, acceptable, and likely to provide benefit for adolescents, and (2) to determine the optimal way to integrate mHealth interventions such as the app into primary care management of adolescent depression. This study focused on adolescents newly diagnosed with depression and anxiety, as we anticipated immediate benefit from using the app while awaiting a therapeutic response from usual care, including antidepressant medication and/or referral for psychotherapy. We hypothesized that the app would be acceptable and easy to use and that use of the app [16] would lead to greater improvement in symptoms for adolescents newly diagnosed with depression and anxiety compared to the wait list control group.

## Methods

### Methodology

Study activities were conducted within the Washington University Pediatric and Adolescent Ambulatory Research Consortium (WU PAARC), part of the WU Institute for Clinical and Translational Science (ICTS). The practice network includes over 100 pediatric care providers from approximately 60 practices in the greater metropolitan St Louis area and serves approximately 175,000 patients. Across the consortium, 25% (approximately 44,000) of the patients are publicly insured (eg, Medicaid) and 28% (approximately 50,000) are Black. Participation was restricted to patients and PCPs from 11 practices from the practice-based research network (PBRN) who had completed a 12-month quality improvement (QI) initiative targeted at improving care for adolescents with depression [37], specifically by establishing systems of care that follow the current national guidelines [12,13]. Study activities included a small pilot randomized trial to gather data from teens and their parent and semistructured interviews with PCPs. The study was conducted from October 2020 to April 2021, encompassing the second wave of COVID-19 infections in the region, which limited or at times completely shut down in-person clinical care delivery during the study period.

### Ethics Approval

The study was approved by the Washington University Institutional Review Board (202005103). Parents or legal guardians provided informed consent, and adolescents provided informed assent to participate in the study. The consent/assent process was initiated by phone due to pandemic-related barriers to in-person participation. Formal consent/assent was conducted using a Research Electronic Data Capture (REDCap)–based electronic consent form. Following a prescreening phone call, the parents or legal guardians of potential adolescent participants received an email with a unique link to review the informed consent form online. After the research team explained the study and answered any questions, parents or legal guardians and potential participants clicked an “agree” button, which was accompanied by text indicating that by clicking the button, they were providing consent/assent to participate. They were then asked to type their full name, which served as their electronic signature confirming consent/assent. Upon completion of the form, participants were presented with the option to download an electronic copy of the executed form. A digital copy was also emailed to each participant’s parent or legal guardian. Participating parents or guardians and adolescents each received up to US $40 as reimbursement for their time to complete study assessments ($5 to $10 per survey).

### Randomized Pilot Study

Participants were randomized 1:1 for 12 weeks either to a wait list control or to the app intervention. Time points for clinical assessments were based on clinical care guidelines (baseline, 4, 8, and 12 weeks) [13], with the primary analysis at 4 weeks. The primary outcome was depression symptom severity on the 9-item Patient Health Questionnaire (PHQ-9). Secondary outcomes included anxiety symptom severity assessed with the 7-item Generalized Anxiety Disorder questionnaire (GAD-7), which uses scoring similar to the PHQ-9 to indicate the frequency and severity of anxiety symptoms, and mental health self-efficacy assessed with the Mental Health Self-Efficacy Scale (MHSES). All patients in the intervention and wait list control groups received usual care for their depression and anxiety from their PCP. Participants in the control group were offered use of the app at the end of the 12-week study period.

### Intervention

W-GenZ delivers evidence-based therapeutic elements via brief “conversations” with a fully automated, relational conversational agent named Woebot. Powered by natural language processing and machine learning techniques, the brief, self-guided intervention draws from CBT, interpersonal psychotherapy for adolescents (IPT-A), and some elements of dialectical behavior therapy (DBT), tailoring the conversation to the present situation to help the adolescent develop emotion regulation skills in the context of their everyday life for the problem at hand. The user experience is centered around mood tracking and goal-oriented, tailored conversations. Woebot checks in with the user, and depending on the user’s reported mood or desire to work on a problem or learn something new, Woebot will offer and guide the user through CBT-based psychoeducation and tools, tailored to the reported need in that moment. Daily push notifications prompt users to check in. Using proprietary neurolinguistic programing and artificial intelligence, the platform designs a personalized program to meet the user’s needs in real time. The app conforms with safety recommendations from the American Psychiatric Association (APA) and American Medical Association (AMA) [38,39]. As part of the app’s onboarding process, users are provided with the privacy policy, theoretical underpinning, targeted difficulties, expected results, research findings, and other information requested by potential young users [18]. Safety features include informing the user they are talking to a robot and not a real person, reminding them that the app is not a crisis intervention for suicidal ideation, encouraging them to seek additional support if they are feeling unwell, and providing helplines for assistance. The safety net protocol detects concerning language, confirms it with the user, and provides a local suicide crisis hotline number as needed.

### Study Population

Adolescents were eligible for inclusion if they were 13 to 17 years old and had a new diagnosis of depression and anxiety in the past 3 months, as reported by their parent or legal guardian. Adolescents were excluded if their parent reported any lifetime history of severe depression, substance use disorder, psychotic illness, obsessive compulsive disorder, posttraumatic stress disorder, panic disorder, or specific phobias, as this could indicate that the current reported episode was a more complicated or severe presentation of depression or that the current episode was recurrent. Those who reported a psychiatric hospitalization in the previous month, were not accompanied by a guardian to the diagnostic visit, did not have access to a mobile device (cell phone/tablet) for regular use, and were unable to read and write English were excluded.

### Recruitment Procedures

Five of the 11 eligible practices referred patients to the randomized controlled trial (RCT). Potential participants were informed about the study via a flyer distributed by their PCP at the diagnostic visit or within the first 3 months after diagnosis. For those interested in participation, the parent completed the online eligibility screen. If the adolescent was eligible, the parent and adolescent reviewed the electronic consent document and had a telephone conversation with a member of the study team to review participation requirements and answer questions.

**Figure 1:**
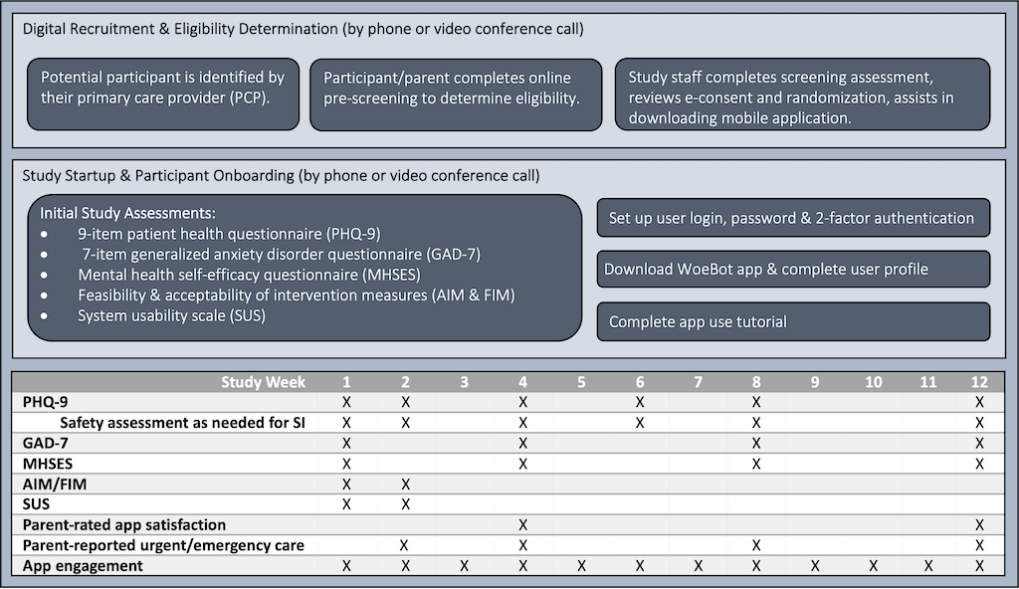
Flowchart indicating the study procedures and schedule of events. AIM: Acceptability of Intervention Measure; FIM: Feasibility of Intervention Measure; GAD-7: 7-item Generalized Anxiety Disorder questionnaire; MHSES: Mental Health Self-Efficacy Scale; PHQ-9: 9-item Patient Health Questionnaire; SUS: System Usability Scale.

If the family decided to participate, the adolescent and 1 parent signed the electronic consent and completed baseline web-based surveys via REDCap. Subsequently, the adolescent was randomized using REDCap [40]. Those randomized to the intervention received a link and a unique password to download the intervention app on their mobile device, proceed through the enrollment screens, and initiate the program. All participants were followed for 12 weeks with assessments, as indicated in Figure 1. After data collection was completed, participants’ medical charts were reviewed to confirm any additional mental health diagnoses and record their initial treatment plan.

### Measurement

In both study groups, study surveys were administered to adolescents and parents through a secure digital link to a REDCap survey. For each survey, participants were directed to a URL via email or text, with up to 2 push notification reminders. Time points for the primary (4 weeks) and safety (2 and 4 weeks) outcomes were selected based on the practice guidelines for PCPs managing adolescent depression [13]. If the measurement was not completed within 1 week, participants received a phone call from a study team member to assess study engagement. The surveys varied in duration and took 5 to 20 minutes to complete. Gift cards were provided to compensate participants for their time to complete data collection and were distributed immediately upon survey completion (up to US $40). Study design and assessments are indicated in Figure 1.

Depression symptoms were assessed using the PHQ-9 [41] modified for use in adolescents (PHQ-A) [42]. The PHQ-A also consists of 9 questions, but with wording changes to accommodate an adolescent reading level, and was used to screen for adolescent depression according to Diagnostic and Statistical Manual of Mental Disorders fifth edition (DSM-5) criteria. Although the unmodified version has good validity and reliability in adolescent populations, the PHQ-A has modified the wording of the questions so that it can be entirely self-administered in individuals with a grade 6 reading level. The overall PHQ-9 score ranges from 0 to 27, with individual item symptom severity scores of 0, 1, 2, and 3, which correspond to “not at all,” “several days,” “more than half the days,” and “nearly every day,” respectively. Overall cumulative scores of 5, 10, 15, and 20 represent cut points for mild, moderate, moderately severe, and severe depression, respectively.

Anxiety symptoms were assessed using GAD-7 [43]. This is a reliable, valid, and responsive screening instrument commonly used in primary care settings [44] and has been extensively studied and validated in diverse adolescent populations, including for screening in primary care settings in Finland [45], China [46], Korea [47], Ghana [48], and Canada [49]. GAD-7 has also been validated in the United States in a clinical population of adolescents with generalized anxiety disorder [50]. Respondents use the same item scoring system as the PHQ-9 to indicate frequency and severity of anxiety symptoms, and a cut point of >10 is used to identify clinical cases of anxiety.

Confidence in self-management of mental health issues was measured using the MHSES [51], which has 6 items that address confidence in managing stress, depression, and anxiety and are scored on a 10-point scale (1: not at all confident, 10: very confident). Ratings are summed for an overall measure of self-efficacy (range 6 to 60), with higher scores indicating more self-efficacy.

Safety was assessed at 2, 4, 8, and 12 weeks. Parents were asked to report any hospitalizations or ED visits made by their child for depression/anxiety-related problems in the preceding 2 weeks up to 1 month.

### Utilization, Acceptability, and Feasibility

Deidentified aggregate app usage data were obtained from the app developer. Metrics included aggregate descriptive summaries of engagement metrics, including total number of check-ins, days in app, and messages sent during the 4-week intervention period. Each conversation with the app is tailored to the users’ reported needs at the moment and thus necessarily varies in length and composition. Nonetheless, these metrics are provided as examples to demonstrate various ways that users engaged with the app’s offerings.

The acceptability and feasibility of using the intervention was assessed after 2 weeks from the perspective of the adolescents (n=10, 56%) and their parent or adult legal guardian (n=10, 44%) randomized to the intervention group of the RCT. Parents completed the 4-item Acceptability of Intervention Measure (AIM) and the 4-item Feasibility of Intervention Measure (FIM) developed by Weiner et al [52]. To aid understanding by the 13- to 17-year-old adolescent participants, only 1 item from each of these measures was used.

Adolescents assessed the usability of the app using 5 items from the 10-item System Usability Scale (SUS) [53] that includes statements about the effectiveness, efficiency, and satisfaction with use (score range 5 to 25). For the AIM, FIM, and SUS, respondents used a 5-item response scale (1: completely disagree, 5: completely agree) to indicate their agreement with item statements, and a summary score was created. For the AIM and FIM, higher scores indicated greater acceptability and feasibility [52,54].

Acceptability and feasibility for PCPs were assessed by semistructured interviews (described in the subsequent section).

### Safety

For all participants, an additional layer of safety monitoring was provided by the study team through surveillance of the digital PHQ-9 assessment at baseline, 2, 4, 6, 8, and 12 weeks. Parents provided consent for communication between the study team and the PCP for this purpose. When the PHQ-9 flagged suicidal ideation, an email alert was sent to the study team, and the PI then contacted the participant’s PCP to share information and discuss next steps regarding assessment of suicide risk. The principal investigator (PI; author JG) summarized the relevant information and alerted the psychiatrist. Management options included (1) the PCP electing to alert the family to the situation and complete additional suicide risk assessment themselves or (2) the PCP requesting further assessment be completed by the pediatric study psychiatrist, who then conducted additional assessment with patients and their legal guardians over the phone and provided a brief written assessment and recommendations back to the PCP. The PCP also had the option of further discussing the case with the study psychiatrist by phone. The PCP’s preferred management approach was prioritized, and the psychiatrist provided further assessment by phone and offered treatment recommendations to the PCP when requested using an evidence-based telephone consultation model [55].

### Statistical Analyses

Summary statistics are reported as percentages for categorical variables and mean and SD for continuous variables. We evaluated the Cohen *d* effect size and 95% CIs on the mean PHQ-9, GAD-7, and MHSES scores achieved by treatment group at the primary end point of 4 weeks. All statistical analyses were completed using SAS software version 9 (SAS Institute Inc).

### PCP Interviews

We completed semistructured interviews with PCPs to better understand current attitudes toward and practices related to recommending CBT to pediatric patients as well as toward the use of mobile health applications for delivering behavioral mental health interventions to youth.

PCPs who participated in the PBRN quality improvement (QI) initiative were eligible to participate in the interviews, and all received an email invitation. Interviews were conducted concurrently with the pilot RCT portion of the study between October 2020 and April 2021. A total of 13 PCPs from the 11 participating clinics completed a 30-minute virtual video interview with the PI, who was also the PBRN director. She used a semistructured interview guide to ask PCP about their use of apps as part of a treatment plan to improve physical and mental health, how they found out about apps to recommend, and positive and negative features of the app considered when selecting an app to recommend. After these general questions, participating PCPs were provided with some promotional material about the intervention app that included a picture of the chatbot (without its name) and information about how it interacts with the user. They were asked about their first impressions, how likely they were to recommend it, and for which patients and when in the treatment course it would likely be most useful. Interviews were continued until thematic saturation was achieved. All interviews were digitally recorded and transcribed verbatim. Participants received a US $50 gift card for participation.

### Qualitative Analysis of PCP Interviews

Transcripts were analyzed using an inductive coding approach based on pragmatic-variant grounded theory [56]. Authors JG and GN independently reviewed interview transcripts for emergent themes in PCP perspectives on the use of mobile applications to augment physical and mental health care.

## Results

### Pilot RCT

**Figure 2:**
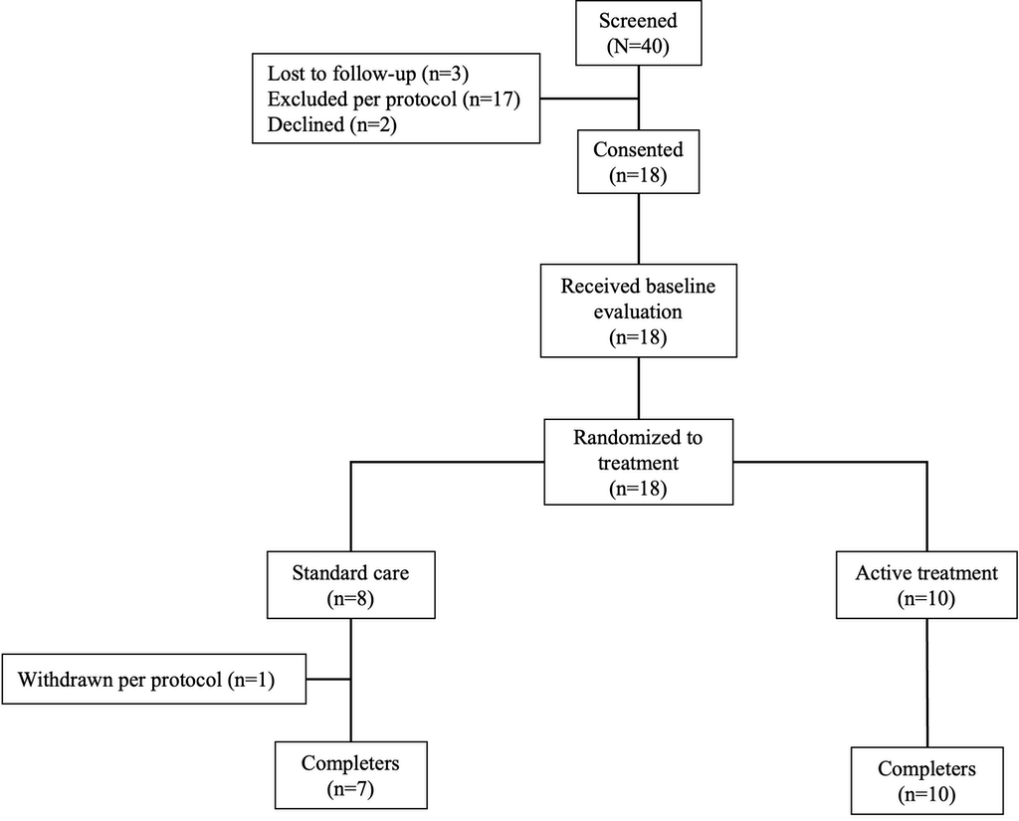
Participant Disposition.

Figure 2 shows participant flow through the pilot RCT. Of the 40 families that completed the eligibility screening for the RCT, 17 (43%) were ineligible, 2 (5%) declined to participate, 3 (8%) were lost to follow-up, and 18 (45%) were randomized to the intervention (10/40, 25%) or wait list control (8/40, 2%). Chart review revealed the following diagnoses: depression (8/18, 44%), depressive symptoms (2/18, 11%), anxiety (16/18, 89%), stress-related headaches (1/18, 6%), and functional abdominal pain and adjustment disorder (1/18, 6%). A review of the notes and baseline PHQ-9 and GAD-7 scores resulted in a sample of 17 for the analyses, with 10 (59%) that had both depression and anxiety 7 (41%) that had anxiety alone. One participant originally randomized to the wait list control group was excluded from analyses following chart review based on the chart notation “episodic tension headaches due to stress” and baseline measures (PHQ-9=5, GAD-7=6), as they were deemed to be ineligible (no diagnosis of depression or anxiety in their chart and no evidence of moderate disease).

### Participant Characteristics

The participating parent was typically the mother (16/17, 94%). Participating teens are described in Table 1 and did not differ by treatment group. They were predominantly female (15/17, 88%), White (15/17, 88%), and had family work-related health insurance (15/17, 88%). Initial treatment plans did not differ between study groups and included CBT (5/17, 29%), unspecified counseling, 15/17, 88%), and SSRI medication (11/17, 65%). By self-report, 7 (41%) teens had previously received at least 1 counselling session for depression or anxiety.

**Table 1:** Randomized Controlled Trial (RCT) participant baseline characteristics.

Characteristic		Total (N=17)	Intervention (N=10)	Wait list control (N=7)
**Sex, n (%)**				
	Male	1 (5.9)	1 (10)	0 (0)
	Female	15 (88.2)	9 (90)	6 (85.7)
	Missing	1 (5.9)	0 (0)	1 (14.3)
	Age in years, mean (SD)	14.7 (1.7)	14.7 (1.7)	14.8 (1.7)
**Race, n (%)**				
	White	15 (88.2)	8 (80)	7 (100)
	Mixed	2 (11.8)	2 (20)	0 (0)
**Ethnicity, n (%)**				
	Non-Hispanic/Latino	17 (100)	10 (100)	7 (100)
**Health Insurance, n (%)**				
	Private insurance	15 (88.2)	9 (90)	6 (85.7)
	Medicaid	2 (11.8)	1 (10)	1 (14.3)
**Living situation, n (%)**				
	Two parents	12 (70.6)	5 (50)	7 (100)
**Diagnosis from chart review, n (%)**				
	Anxiety disorder	17 (100)	10 (100)	7 (100)
	Depressive disorder	10 (58.8)	5 (50)	5 (71.4)
**Initial treatment plan from chart review, n (%)**				
	SSRI^a^	11 (64.7)	6 (60)	5 (71.4)
	Other medication(s)	0 (0)	0 (0)	0 (0)
	CBT^b^	5 (29.4)	3 (30)	2 (28.6)
	Unspecified counseling	15 (88.2)	8 (80)	6 (85.7)
	Referral to psychiatry	2 (11.8)	1 (10)	1 (14.3)
**Measures at Baseline, mean (SD)**				
	PHQ-9^c^	12.1 (5.3)	10.1 (3.9)	14.9 (6)
	GAD-7^d^	12.8 (4.9)	11 (5)	15.3 (3.5)
	MHSES^e^	30.9 (10.2)	32.4 (10.1)	28.9 (10.9)

^a^SSRI: selective serotonin reuptake inhibitor.

^b^CBT: cognitive behavioral therapy.

^c^PHQ-9: 9-item Patient Health Questionnaire.

^d^GAD-7: 7-item Generalized Anxiety Disorder questionnaire.

^e^MHSES: Mental Health Self-Efficacy Scale.

### Pilot RCT Outcomes

**Figure 3:**
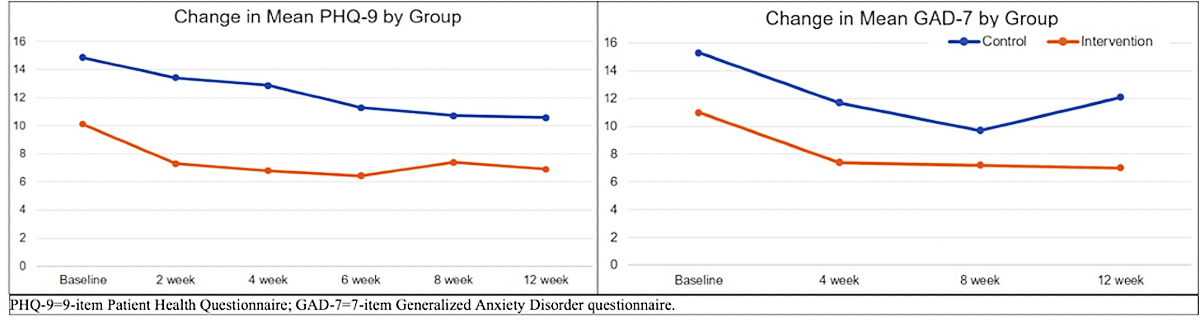
Change in mean PHQ-9 and GAD-7 scores by treatment group over 12 weeks. GAD-7: 7-item Generalized Anxiety Disorder questionnaire; PHQ-9: 9-item Patient Health Questionnaire.

Table 2 and Figure 3 show the overall study results. The small sample size precluded meaningful comparisons between study groups. However, large effect sizes were observed between groups on depression symptom severity score at 4 weeks, with the intervention group showing greater improvement on each scale than the wait list control group. Specifically, mean PHQ-9 scores at 4 weeks decreased by 3.3 units in the intervention group (representing a transition from moderate to mild symptom severity categories) and 2 units in the wait list control group (no shift in symptom severity category). In subgroup analyses among the 10 participants diagnosed with depression, PHQ-9 scores similarly decreased by 3 units in the intervention group (11.8 to 8.8; from moderate to mild severity categories) versus 1 unit in the wait list control group (15 to 14; no shift in symptom severity category).

**Table 2:** Primary and secondary study outcomes at 4 weeks.

Outcome, mean (SD)	Intervention group	Wait list control group	95% CI	Cohen *d*
PHQ-9^a^	6.8 (6.9)	12.9 (5.4)	−12.9 to 0.7	.98
GAD-7^b^	7.4 (6.5)	11.7 (4.8)	−10.6 to 2	.75
MHSES^c^	38.7 (8.4)	31.7 (11)	−3.4 to 17.3	.71

^a^PHQ-9: 9-item Patient Health Questionnaire.

^b^GAD-7: 7-item Generalized Anxiety Disorder questionnaire.

^c^MHSES: Mental Health Self-Efficacy Scale.

We also compared remission in depressive symptoms (PHQ-9 <5) at 4 and 12 weeks in patients with a baseline PHQ-9 >9 [57] in the subgroup diagnosed with depression. The percentage of participants achieving remission at both time points seemed to favor the active intervention, at 67% (2/3) and 0% (0/5) at 4 weeks and 50% (1/2) and 20% (1/5) at 12 weeks, respectively.

### Safety and Satisfaction With Care

When completing the PHQ-9 on one of the 6 adolescent surveys, 10 (59%) of participants, of which 4 (40%) were in the intervention group and 6 (35%) were in the wait list control group, triggered at least 1 alarm to assess for suicidal ideation. During study participation, 4 (24%) of the participants had 1 alert, 4 (24%) had 3, and 2 (12%) had 6. These patients were managed by their PCP with support from a pediatric research psychiatrist, who interviewed all 4 (24%) participants by phone, discussed management with the legal guardian (who was the mother in all cases), and facilitated referral of 2 (12%) participants to a child psychiatrist for ongoing clinical management. One parent from the intervention group reported at week 12 that their teen was seen in an ED and discharged to home. Parental satisfaction with care did not differ between study groups.

### Acceptability, Feasibility, and Usability for Adolescents and Parents

Adolescents found the app to be (1) acceptable, with a total of 8 (80%) agreeing or completely agreeing with the statement “I like using the app”; (2) feasible, with 7 (70%) agreeing with the statement “using the app in the treatment of depression seems possible”; and (3) usable (mean usability score 21.4, SD 1.7, possible range 5 to 25). The mean parental scores for acceptability and feasibility were 16.6 (SD 1.8) and 17 (SD 1.5), respectively (possible range 1 to 20).

### Acceptability and Feasibility for Providers

Potential advantages for learning CBT skills via an app compared with face-to-face training identified by provider participants included ease/immediacy of access to evidence-based behavioral therapy, increased sense of anonymity or reduction in perception of stigma against mental health concerns, reduction in school absences to attend appointments, no need for transportation to and from a therapist’s office, and reduction of out-of-pocket costs. Although providers acknowledged that an app might reduce common barriers to engagement in therapy, they voiced several concerns about using an app to augment clinical recommendations. Concerns commonly involved efficacy of the app, the potential safety risks of missing reports of suicidal ideation, overreliance on technology and reduced self-efficacy for adaptive help-seeking, increased screen time, and privacy and confidentiality of sensitive information.

Providers generally felt that an app to deliver CBT and positive psychology could be useful for teens with anxiety and mild-to-moderate depression, particularly early in the course of illness, and saw the app as being able to bridge the gap between diagnosis and obtaining an appointment with a therapist or the onset of therapeutic effects of prescribed antidepressant medications—both of which can take several weeks. Due to acute safety concerns, they recommended against using an app in teens with active suicidality or self-harm. They felt these patients needed closer clinical monitoring and were unlikely to use the app because of their low motivation to adopt new behaviors.

PCP participants suggested that the best time to introduce a CBT app to patients and families as part of the treatment plan would be at the time of diagnosis. Several suggested that usage might be improved if the pediatrician introduced the app during an appointment and showed them how to download and use it (eg, by saying to the patient, “Here’s what I’d like you to start doing…”). In summary, a CBT app was acceptable to these pediatric PCPs, and they suggested that it would be feasible to use in teens with mild/moderate depression or anxiety.

### App Utilization

Participant app use during the intervention period (collected via the app) included days of app use, number of check-ins, and number of messages sent, lesson and tools use rates, lesson acceptability ratings indicated on a binary scale (ie, a thumbs up or thumbs down emoticon), and mood impact after tool utilization (ie, feeling same, better, or worse after completion). In-the-moment emotional state was reported through emoji selection with a default menu of 19 total moods, including options for negative (angry, sad, and anxious), positive (happy and content), and average mood (okay), with an additional ability to type in free text emotion words and self-selected emoji expressions. 

Over the course of the intervention from baseline to 4 weeks, participants’ app use averaged a mean of 6 (SD 6.9) days, with 55 (SD 7.14) mood check-ins, and 313.17 (SD 447.30) sent messages, with the use of 13.63 (SD 4.5) psychoeducational lessons and 9 (SD 14.91) tools. All completed psychoeducational lessons were rated positively (thumbs up). Over two-thirds (7/10, 69%) of participants reported feeling better after using the app, with 25% (3/10) and 1% (1/10) reporting feeling the same or worse, respectively.

## Discussion

In this pragmatically designed, randomized pilot and feasibility study, we demonstrated the preliminary acceptability and feasibility of augmenting initial depression treatment in adolescents with CBT delivered via an evidence-based chatbot intervention. Although the sample size needed to detect between-group differences was not achieved, we observed a trend toward improvement in depressive symptoms with the app over a wait list control condition. Moreover, we observed that the detection and management of suicidal ideation in depressed adolescents was safely enhanced by use of the app in concert with PCP-based clinical care and telephonic consultation with a child psychiatrist. Finally, we found that using this app to address depression and anxiety as part of an overall treatment plan developed in collaboration with the treating PCP was acceptable to the adolescents, their parents, and their PCPs. These findings suggest that this app should be further evaluated for use in the primary care management of adolescents with depression and anxiety.

Adolescents visit their PCP 2 to 3 times per year on average and commonly report feeling most comfortable disclosing mental health concerns and obtaining mental health care in the primary care setting [14]. A recent review to assess the efficacy and acceptability of cell phone apps to support mental health management in adolescents found high levels of adherence, but few evaluative studies employed quantitative assessment of these process measures, which are key for guiding real-world implementation. This small pilot study provided additional lessons regarding real-world clinical implementation of mobile health interventions in pediatric primary care. For example, the frequency of suicidal ideation reporting (as assessed by question 9 of the PHQ-9) decreased over the course of study participation, even when the severity of other depressive symptoms remained in a clinically significant range. Although this study was not designed to detect differences between groups on suicidality, we observed a reduction in self-reported suicidal ideation that appeared to favor the active treatment group. This is particularly relevant in the delivery of mental health care to adolescent populations, who may feel more comfortable sharing such information anonymously through an app than with a trusted adult or health care provider.

Our results are subject to important limitations. First, despite using a fully remote study design and a well-established PBRN during a period of high mental health need, recruitment was challenged by the circumstances of the COVID-19 pandemic and the reduced access to and utilization of primary care resources, especially early on in the pandemic. Although the hybrid study design is important for piloting the intervention in the patient population of interest, the results must be interpreted with caution. Few conclusions can be drawn about intervention effectiveness due to the small sample size and the racial and geographic homogeneity and relative affluence of the enrolled participants, who were mostly female, White, and privately insured. The ability to detect differences between treatment groups may have been further limited by the concurrent use of antidepressant medications in some participants. The results regarding implementation effectiveness are subject to additional limitations, as the PBRN and PBRN providers in this study are not representative of the broader population of pediatric primary care clinics or providers in the United States [16]. Moreover, our safety approach of providing case review and rapid, individualized review and treatment planning with a child psychiatrist may also have contributed to the lack of separation between treatment groups, as everyone in the study received high-quality clinical care and close follow-up for suicidality reported on the PHQ-9.

The fully remote nature of the trial, which was conducted during critical periods in the COVID-19 pandemic, when child and adolescent mental health needs were most critical, was both a limitation and a strength. Additionally, this is the first study we are aware of that employed digital assessment and remote methods for managing suicidality in adolescents. The concern that suicidality will be exacerbated or missed with digital interventions has been a major barrier to offering these treatments to youth. The method we used to ensure safety (phone consultation with a child psychiatrist within 24 hours of reporting clinically significant suicidal ideation) could not have disguised symptom severity in the wait list control group, since we were responsive to all alerts. Another major strength of this pilot study was evaluating the feasibility of implementation in a real-world primary care setting. Although this study does not provide sufficient evidence to support the use of apps in primary care settings for teens with mental health problems [21], the results presented in this paper provide promising evidence that preliminarily support the feasibility of using a CBT-based chatbot technology to supplement mental health treatment of adolescents with mild-to-moderate depression cared for in the primary care setting. Additional studies are needed to further evaluate the effectiveness of both the intervention and implementation in real-world primary care settings and in patient populations that include underrepresented populations and prioritize socioeconomically disadvantaged and rural communities, where access to high quality medical and mental health care are extremely limited.

## Abbreviations

AIM: Acceptability of Intervention Measure
AMA: American Medical Association
APA: American Psychiatric Association
CBT: cognitive behavioral therapy
DBT: dialectical behavior therapy
DSM-5: Diagnostic and Statistical Manual of Mental Disorders fifth edition
ED: emergency department
FIM: Feasibility of Intervention Measure
GAD-7: 7-item Generalized Anxiety Disorder questionnaire
ICTS: Institute for Clinical and Translational Science
IPT-A: interpersonal psychotherapy for adolescents
mHealth: mobile health
MHSES: Mental Health Self-Efficacy Scale
PBRN: practice-based research network
PCP: primary care provider
PHQ-9: 9-item Patient Health Questionnaire
PHQ-A: 9-item Patient Health Questionnaire modified for Adolescents
PI: principal investigator
QI: quality improvement
RCT: randomized controlled trial
REDCap: Research Electronic Data Capture
SNRI: serotonin-norepinephrine reuptake inhibitor
SSRI: selective serotonin reuptake inhibitor
SUS: System Usability Scale
WU PAARC: Washington University Pediatric and Adolescent Ambulatory Research Consortium
